# Enhanced tumor cell killing by ultrasound after microtubule depolymerization

**DOI:** 10.1002/btm2.10233

**Published:** 2021-06-11

**Authors:** Aditi Singh, Ajay Tijore, Felix Margadant, Chloe Simpson, Deepak Chitkara, Boon Chuan Low, Michael Sheetz

**Affiliations:** ^1^ Mechanobiology Institute National University of Singapore Singapore; ^2^ Department of Pharmacy Birla Institute of Technology and Science Pilani India; ^3^ Biochemistry and Molecular Biology Department University of Texas Medical Branch Galveston Texas USA

**Keywords:** apoptosis, cancer therapy, mechanical forces, microtubules, Piezo1, ultrasound

## Abstract

Recent studies show that tumor cells are vulnerable to mechanical stresses and undergo calcium‐dependent apoptosis (mechanoptosis) with mechanical perturbation by low‐frequency ultrasound alone. To determine if tumor cells are particularly sensitive to mechanical stress in certain phases of the cell cycle, inhibitors of the cell‐cycle phases are tested for effects on mechanoptosis. Most inhibitors show no significant effect, but inhibitors of mitosis that cause microtubule depolymerization increase the mechanoptosis. Surprisingly, ultrasound treatment also disrupts microtubules independent of inhibitors in tumor cells but not in normal cells. Ultrasound causes calcium entry through mechanosensitive Piezo1 channels that disrupts microtubules via calpain protease activation. Myosin IIA contractility is required for ultrasound‐mediated mechanoptosis and microtubule disruption enhances myosin IIA contractility through activation of GEF‐H1 and RhoA pathway. Further, ultrasound promotes contractility‐dependent Piezo1 expression and localization to the peripheral adhesions where activated Piezo1 allows calcium entry to continue feedback loop. Thus, the synergistic action of ultrasound and nanomolar concentrations of microtubule depolymerizing agents can enhance tumor therapies.

## 1 INTRODUCTION

Recent findings highlighted the substantial differences between the mechanical properties of tumor and normal cells indicating altered mechanosensing as an important feature of tumor cells.^1^, ^2^, ^3^ Detailed studies showed that many tumor cells from different tissue origin lack rigidity sensor that is required for proper substrate rigidity sensing.^3^ The rigidity sensors contain several cytoskeletal mechanosensory proteins, for example, tropomyosin 2.1 and myosin IIA. Because rigidity sensors are missing in tumor cells due to depletion of mechanosensory proteins, cells show transformed growth on soft surfaces. However, the restoration of rigidity sensors by expressing mechanosensory protein that are depleted in tumor cells blocks the transformed growth. Conversely, normal cells have rigidity sensors and cannot grow on soft surfaces, but depletion of sensors by cytoskeletal protein depletion can enable transformed growth. Transformation by depletion of rigidity sensors causes changes in the levels of 700–1000 mRNAs,^3^ which indicates that the activation of growth on soft surfaces involves a major change in the cell state. These properties are similar for many tumor cells from different tissues, since the majority of tumor cells lack the rigidity sensors. This has a practical consequence that the treatments inhibiting transformed cell growth inhibit the tumor cell growth. In studies of tumor cell growth, it has been observed that the loss of mechanosensing causes tumor cells to be damaged by mechanical stresses resulting in growth inhibition and apoptosis.^4^, ^5^ For instance, periodic exercise or stretching of tumors in an animal model leads to tumor regression.^6^, ^7^ Moreover, NIH National Cancer Institute lists seven different types of cancer that are inhibited by exercise and another eight tumor types where data are suggestive.^8^ Further, our latest studies of stretch‐ or ultrasound (US)‐mediated tumor cell apoptosis (mechanoptosis) establish the role of calcium‐activated calpains in inducing a mitochondrial apoptotic pathway.^9^, ^10^

Other US treatments of tumors rely on the activation of multiple pathways to cause cell apoptosis and necrosis. For instance, high‐intensity focused US (HIFU),^11^ high‐intensity pulsed US,^12^ and low‐intensity pulsed US^13^ have been used to ablate tumors. Moreover, US has been used in combination with other treatments like hyperthermia, chemotherapy, and sonodynamic therapy to enhance the efficacy of tumor treatment.^14^, ^15^, ^16^ In terms of mechanism of tumor cell killing, US activates oxidative stress, mitochondrial damage, and DNA damage, which stimulates an apoptotic pathway.^17^, ^18^ However, there are health concerns due to hyperthermia causing damage to healthy cells surrounding the target and thus, these US methods have found limited clinical use. Similarly, severe side effects are associated with conventional chemo‐ and radiotherapy, which severely compromises the patient's quality of life. Therefore, in recent years, a premium has been placed on the strategies that minimize the side effects and interestingly, there are no discernable side effects of the low‐frequency US.

We observed that low‐frequency US causes selective tumor cell apoptosis (mechanoptosis) in vitro and in vivo without damaging normal cells.^9^ To further understand the mechanoptosis process, we tested whether cancer cells are more vulnerable to US damage in certain phases of the cell cycle.^19^, ^20^ Tumor cells were pre‐treated with cytostatic concentrations of cell‐cycle phase inhibitors and then exposed to low‐frequency US. Surprisingly, only inhibitors of mitosis increased the US‐mediated apoptosis. When the concentration dependence was measured, we found that tumor cells treated with nanomolar concentrations of microtubule depolymerizing agents (MDAs) (M‐phase inhibitors) followed by US treatment increased mechanoptosis. Further examination of tumor cells revealed that the US treatment alone disrupted microtubules and thus, an even greater microtubule disruption occurred when MDAs and US treatment were combined together. US‐mediated microtubule disruption was caused by calcium‐activated calpain cleavage. The microtubule disruption resulted in enhancing myosin IIA contractility as previously reported.^21^, ^22^ Further, there was contractility‐dependent Piezo1 localization to peripheral adhesions where activated Piezo1 allowed calcium entry creating a positive feedback loop. Simultaneously, calpain initiated the mitochondrial apoptotic pathway to promote mechanoptosis. Thus, US‐mediated tumor cell apoptosis can be enhanced using nanomolar concentration of clinically approved drugs to more effectively damage tumor cells.

## 2 RESULTS

### 2.1 MDAs enhance US‐mediated tumor cell apoptosis

**FIGURE 1:**
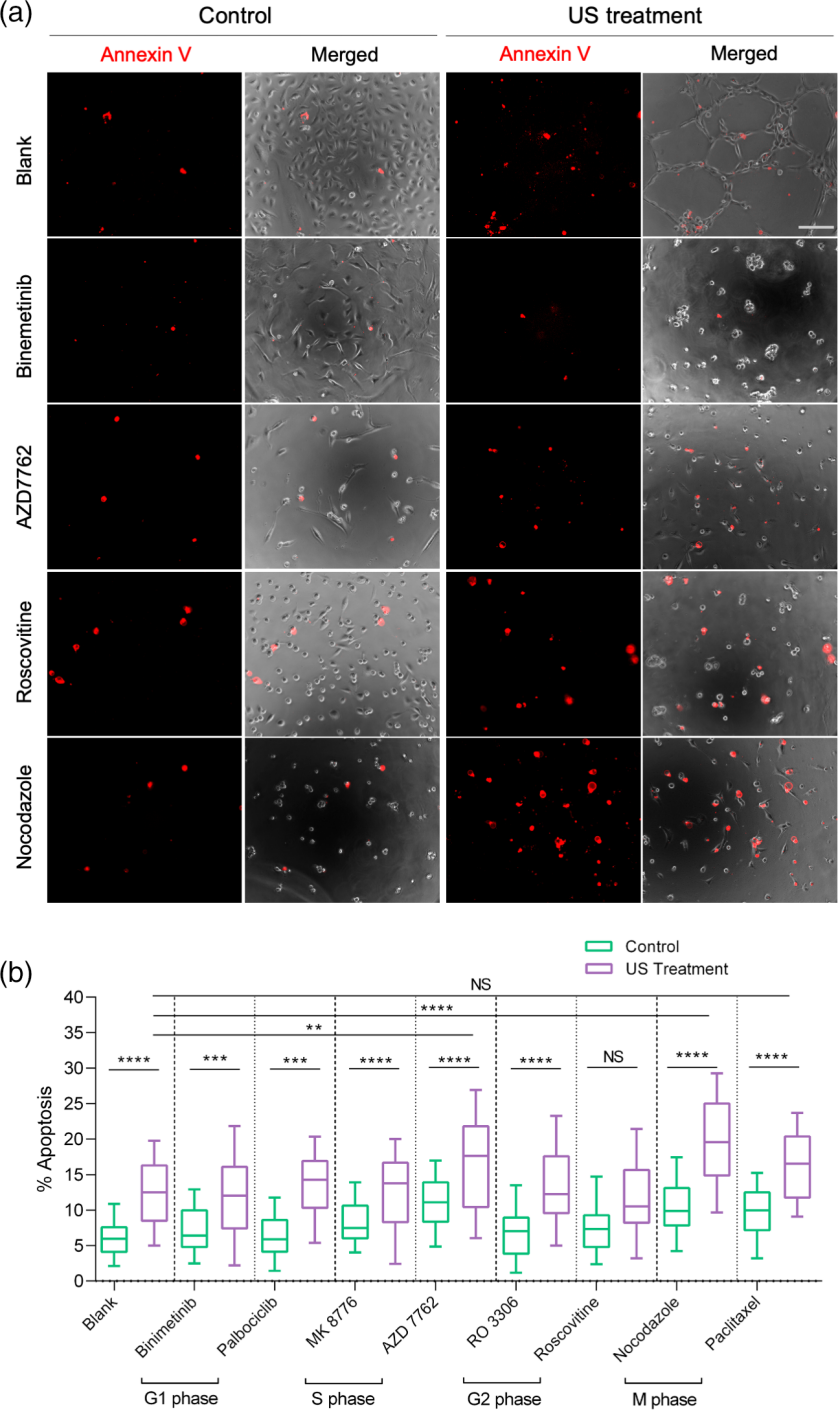
Microtubule depolymerizing agent enhances ultrasound‐mediated MDA‐MB‐231 cancer cell apoptosis. (a) Panels showing annexin V‐stained apoptotic cells in the presence and absence of pharmacological inhibitors at cytostatic concentration, with and without ultrasound treatment, scale bar: 100 μm. (b) Box‐and‐whisker plot displaying level of apoptosis in the presence of pharmacological inhibitors with and without US treatment. *n* > 5000 cells (1500 cells for each condition per experiment), data are representative of four independent experiments. **p < 0.01, ***p < 0.001, ****p < 0.0001

Recently, we observed that many tumor cells from different tissue origin were vulnerable to mechanical stresses and exhibited mechanoptosis upon mechanical activation.^5^, ^10^ We hypothesized that such mechanoptosis was dependent on the cell‐cycle phase and cells were more vulnerable to mechanical stresses when present in a particular cell‐cycle phase. To test the hypothesis, MDA‐MB‐231, a highly metastatic breast carcinoma cell line, was selected. Tumor cells plated on matrigel were treated with concentrations of pharmacological inhibitors that arrest and synchronize the majority cells in specific cell‐cycle phases (Figure S1 and Table S1). Cells grown on matrigel were subjected to low‐frequency US (33 kHz) for 2 h with 7.7 mW/cm^2^ power intensity and a 50% duty cycle. US‐treated tumor cells showed significantly higher apoptosis (13% vs. 6%) compared to the non‐treated cells (Figure 1(a),(b)). When pharmacological inhibitor‐treated tumor cells were further exposed to US, nocodazole‐treated cells (MDA, M‐phase inhibitor) displayed significantly higher apoptosis (20% vs. 10% and 13%) compared to the controls (nocodazole treatment and US treatment, respectively). Surprisingly, paclitaxel‐treated cells (microtubule stabilizing agent, M‐phase inhibitor) did not display an elevated level of apoptosis upon US treatment in comparison to US treatment alone (17% vs. 13%). In addition, S‐phase inhibitor AZD 7762‐treated cells also showed an increase in the cell apoptosis upon US treatment.

To determine if US‐mediated apoptosis was increased by MDAs in other cell lines, metastatic melanoma cell line, A375p, was used (Figure S2). US treatment caused an increase in apoptosis level in melanoma cells (14% vs. 6%) as compared to the non‐treated melanoma cells. Nocodazole‐treated melanoma cells also showed notably high apoptosis upon US treatment (24% vs. 11% and 12%) compared with nocodazole treatment and US treatment, respectively. To test if other MDAs increased US‐mediated apoptosis, MDA‐MB‐231 cells were treated with another clinically approved MDA, vincristine.^23^ Vincristine treatment also enhanced US‐mediated apoptosis in MDA‐MB‐231 cells as compared to the US treatment alone (Figure S3).

To determine if MDAs and US treatment affected normal cells, primary human foreskin fibroblasts (HFF) were exposed to US after being treated with MDAs. Interestingly, there was a negligible apoptosis in both control and treated samples (Figure S4), indicating that normal cells were resistant to the combination of MDA and US treatment. Altogether, results showed that US‐mediated tumor cell apoptosis was enhanced using cytostatic concentration of MDAs without damaging normal cells.

### 2.2 US disrupts microtubules via calpain activation in tumor cells

**FIGURE 2:**
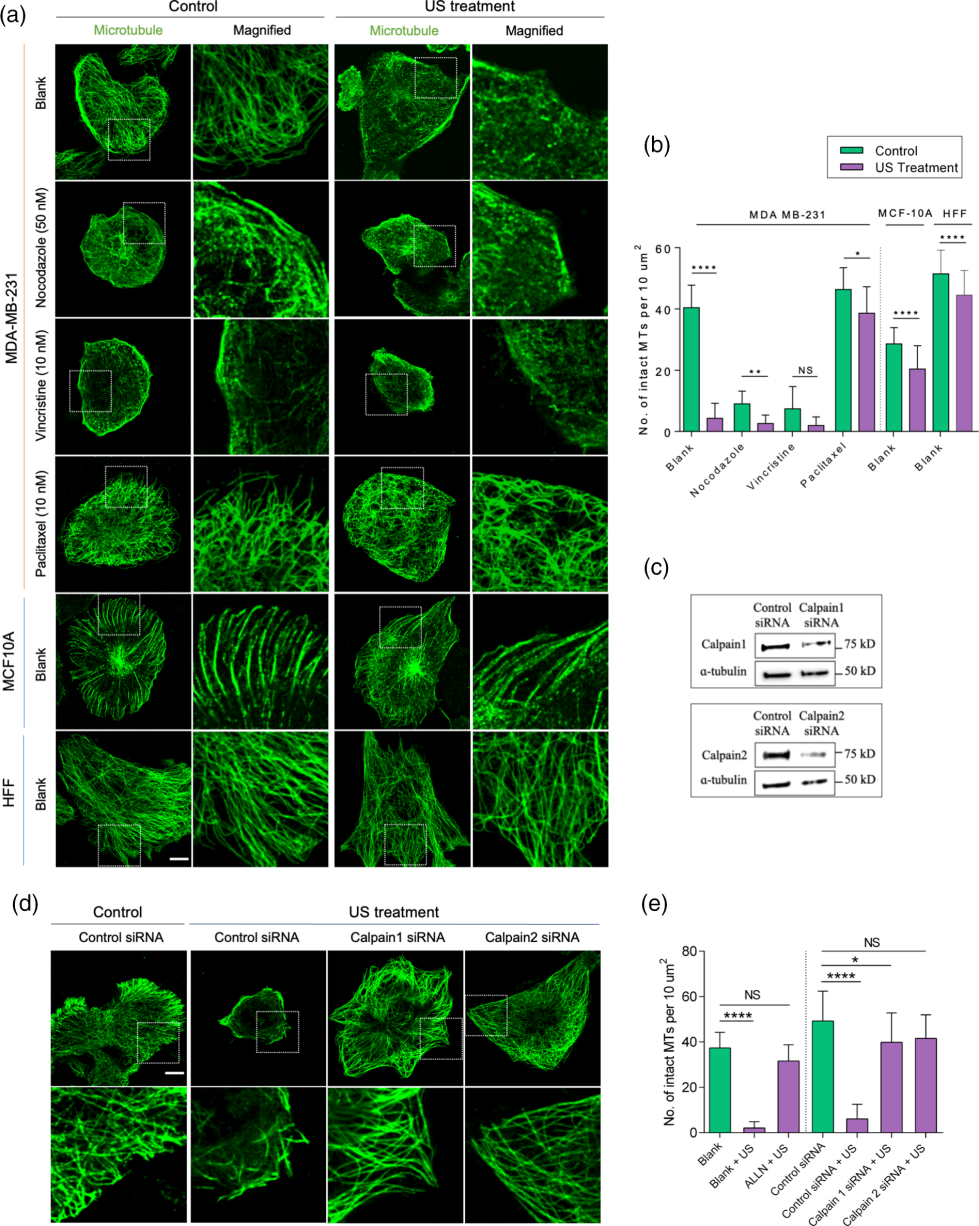
Ultrasound disrupts microtubules by activating calpains in tumor cells. (a) Representative images illustrating microtubule assembly in tumor cells (MDA‐MB‐231) and normal cells (MCF10A and HFF) in the presence of different microtubule targeting agents with and without US treatment, scale bar: 10 μm. (b) Bar diagram demonstrating number of intact microtubules in the defined area in cells under different experimental conditions. *n* > 30 cells, data are representative of two independent experiments. (c) Western blots showing calpain level in control siRNA‐ and calpain siRNA‐treated tumor cells. (d) Panels displaying microtubule assembly in control siRNA and calpain siRNA treated tumor cells with and without US treatment. (e) Bar diagram demonstrating number of intact microtubules in the defined area under different experimental conditions. Data are representative of two independent experiments, *n* > 25 cells. *p < 0.05, **p < 0.01, ****p < 0.0001. In all image panels, scale bar: 10 μm

To investigate the cause of elevated apoptosis using MDAs, we initially examined the effect of US treatment alone and combined MDAs + US treatment on the microtubule assembly of MDA‐MB‐231 cells (Figure 2(a),(b)). Surprisingly, US treatment alone disrupted the microtubule network (eightfold reduction in the number of intact microtubules per 10 μm^2^ in comparison to the non‐treated cells (~40 vs. 5). However, normal cells like epithelial cells (MCF10A, ~28 vs. 20) and fibroblasts (HFF, ~50 vs. 45) showed minimal microtubule disruption upon US treatment, indicating that they were largely unaffected by the US forces. Next, treatment with nocodazole or vincristine caused microtubule disassembly in MDA‐MB‐231 cells. Microtubule quantification results, however, revealed greater extent of microtubule disruption with nocodazole/vincristine + US treatment as compared to drug treatment alone. When MDA‐MB‐231 cells were treated with paclitaxel, the highest number of intact microtubules per 10 μm^2^ (~48) was noticed. Interestingly, paclitaxel‐treated cells upon US treatment exhibited comparatively low microtubule disruption (~38). These observations perhaps explained the reason behind low level of apoptosis in paclitaxel‐treated tumor cells upon US treatment. Altogether, these findings suggested that combination of MDAs + US treatment enhanced the tumor cell apoptosis in part due to increased disruption of microtubules.

Because calcium‐activated calpain 1 and 2 caused the proteolytic degradation of microtubules and microtubule‐associated proteins,^24^, ^25^ we wondered if they were involved in US‐mediated depolymerization of microtubules. To check if the US‐induced microtubule disruption was through calpain activation, we depleted the calpains using siRNA against calpain 1 and 2 (Figure 2(c)). Specific siRNAs against calpain 1 and 2 inhibited the US‐mediated microtubule disassembly (~41–42 vs. 4 microtubules/μm^2^) (Figure 2(d),(e)). Further, these results were confirmed using ALLN treatment (chemical inhibitor of calpain 1 and 2), which inhibited the US‐mediated microtubule disassembly in comparison to non‐treated cells (~38 vs. 2 intact microtubules per 10 μm^2^) (Figures 2(e) and S5). Thus, the US‐mediated mechanical forces disrupted microtubules through calpain activation.

### 2.3 US‐induced microtubule disruption promotes GEF‐H1 release, myosin IIA activity, and adhesion maturation

**FIGURE 3:**
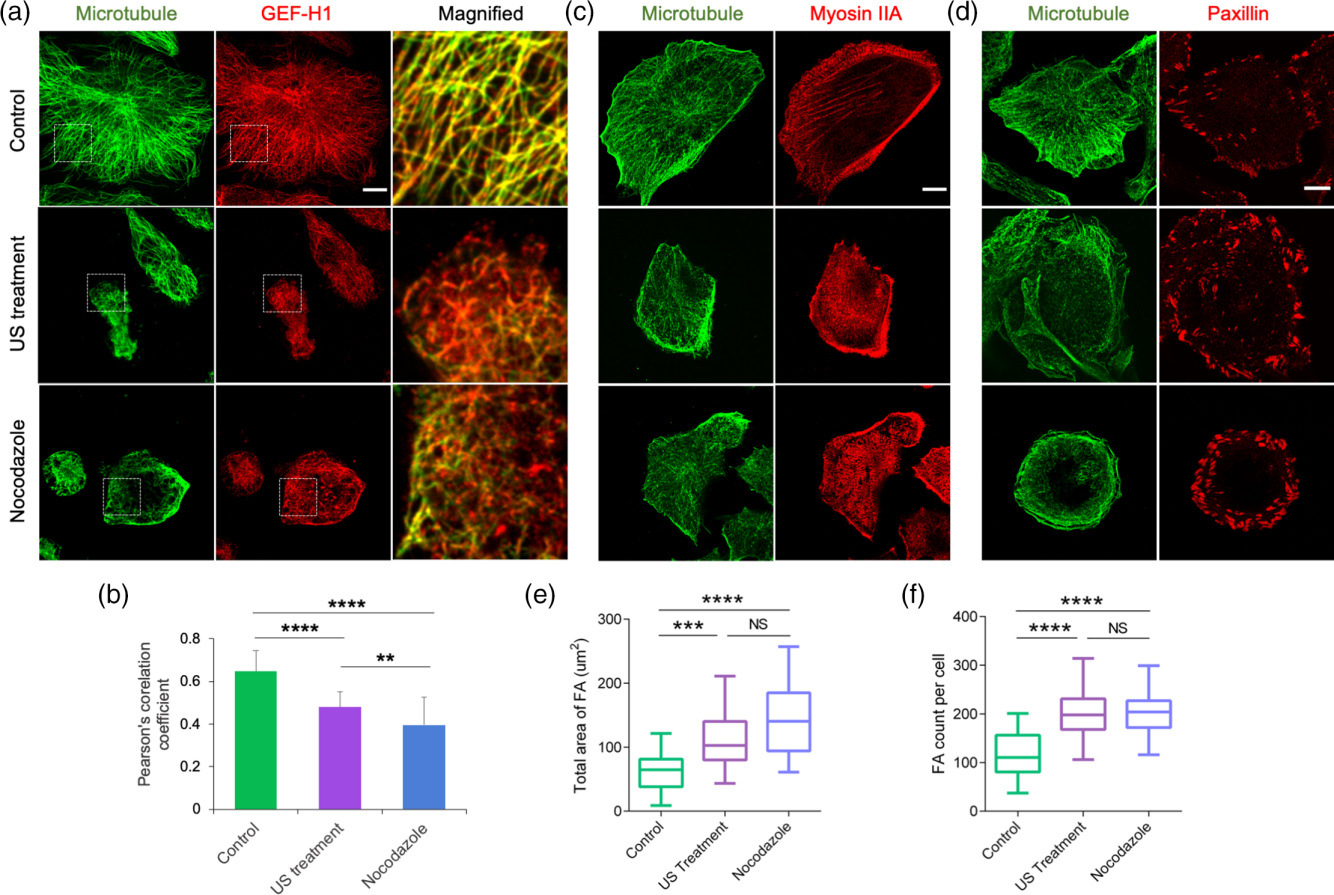
Ultrasound promotes GEF‐H1 release, myosin‐IIA assembly, and adhesion size. (a) Immunofluorescent images showing spatial distribution of microtubules and GEF‐H1 in the control, ultrasound‐ and nocodazole‐treated MDA‐MB‐231 tumor cells. (b) Pearson's correlation coefficient displaying the extent of microtubule and GEF‐H1 colocalization under different experimental condition, *n* = 25 cells from two independent experiments. (c) Representative images showing spatial distribution of microtubules and myosin IIA in the control, ultrasound‐ and nocodazole‐treated cells. (d) Representative images displaying microtubules and paxillin in the control, ultrasound‐ and nocodazole‐treated cells. (e,f) Box‐and‐whisker plot indicating total area of adhesions per cell and number of adhesions per cell respectively in the control, ultrasound‐ and nocodazole‐treated cells, *n* = 25 cells. Whiskers extend from the minimum to maximum values, the box extends from the 25th to the 75th percentile and the line within the box represents the median. **p < 0.01, ***p < 0.001, ****p < 0.0001. In all image panels, scale bar: 10 μm

Microtubule disruption promoted RhoA‐mediated contractility through the release of guanine nucleotide exchange factor (GEF‐H1)^21^, ^22^ from intact microtubules. Because US caused microtubule disruption, it should have altered GEF‐H1 distribution in tumor cells. In non‐treated cells, GEF‐H1 was associated with the microtubules (Figure 3(a)), but after US treatment that disrupted microtubules, it was largely cytoplasmic. Likewise, nocodazole‐treated cells with disrupted microtubules had a diffuse distribution of GEF‐H1. Our observations were supported by a Pearson's correlation coefficient that measured the strength of association between microtubules and GEF‐H1 (Figure 3(b)). Significant reduction in the coefficient value occurred with US and nocodazole treatment as compared to the non‐treated cells.

Next, we investigated the effect of GEF‐H1 release on myosin IIA assembly. In non‐treated cells, myosin IIA assembly was at the cell periphery (Figure 3(c)). In contrast, US‐ and nocodazole‐treated cells demonstrated pronounced myosin IIA assembly throughout the cell. Since myosin IIA directly modulated focal adhesion (FA) dynamics, we assessed the spatial distribution of FA under different conditions. Consistent with the above findings, US and nocodazole treatment caused an increase in FA size and numbers in comparison to non‐treated cells (Figure 3(d)–(f)). Collectively, the results indicated that US‐mediated microtubule disruption caused an increase in myosin IIA contractility through GEF‐H1 release.

### 2.4 US‐mediated contractility promotes Piezo1 expression and localization to peripheral adhesions

**FIGURE 4:**
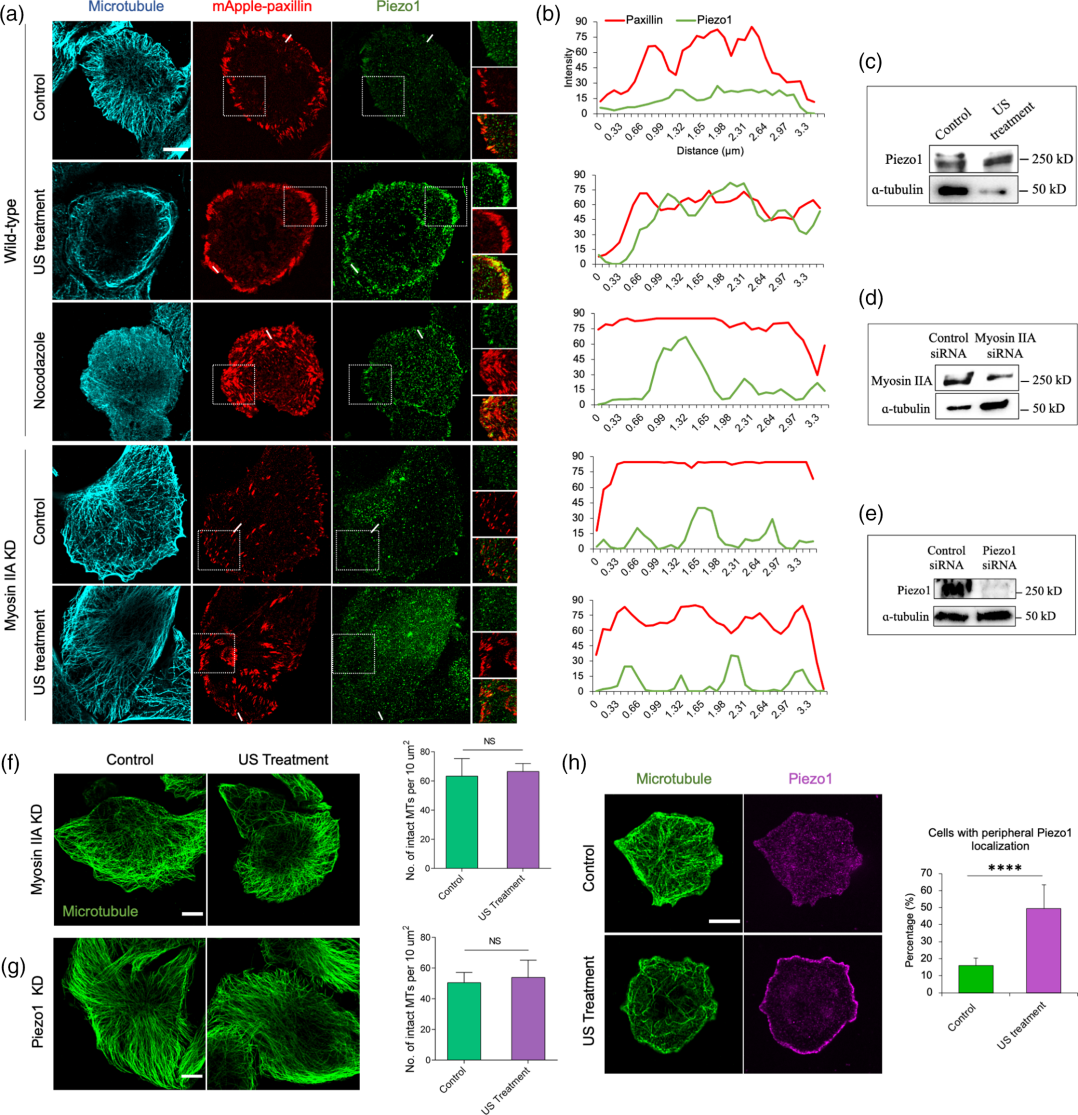
Ultrasound induces Piezo1 expression and localization to peripheral adhesion in MDA‐MB‐231 cancer cells. (a) Fluorescent images showing spatial distribution of microtubule, paxillin, and Piezo1 in the wild‐type (control, ultrasound‐ and nocodazole‐treated) and myosin IIA KD (control and ultrasound‐treated) cells. (b) Corresponding fluorescent intensity plot displaying paxillin and Piezo1 distribution in cells under different experimental condition described in panel a. (c) Western blot measuring the Piezo1 expression level in control and US‐treated cancer cells. (d) Western blot showing myosin IIA expression in control siRNA and myosin IIA KD cancer cells. (e) Western blot showing Piezo1 expression in control siRNA and Piezo1 KD cancer cells. (f) Fluorescent images showing microtubule network in myosin IIA KD cells and bar diagram demonstrating number of intact microtubules with and without US treatment. *n* = 15 cells. (g) Fluorescent images showing microtubule network and bar diagram showing number of intact microtubules in Piezo1 KD cells with and without US treatment, *n* = 25 cells. (h) Representative images illustrating microtubule network and Pieoz1 localization in control and US‐treated cells and bar diagram estimating cell population with peripheral Piezo1 in control and US‐treated cells, *n* > 204 cells. ****p < 0.0001. In all image panels, scale bar: 10 μm

Recent studies have demonstrated myosin IIA contractility‐dependent association of Piezo1 (mechanosensitive calcium channels) with the cell adhesions.^26^, ^27^ Also, our recent findings emphasized the role of Piezo1 in tumor cell mechanoptosis.^9^, ^10^ Therefore, we examined the effect of US treatment on Piezo1 expression and its spatial distribution in tumor cells. The MDA‐MB‐231 cells transiently transfected with mApple‐paxillin were used to determine the adhesion distribution. In non‐treated MDA‐MB‐231 cells, intact microtubules and peripheral adhesions were observed, while Piezo1 was mostly diffuse in the cytoplasm (Figure 4(a),(b)). However, upon US treatment, microtubules were disrupted and there was a distinct Piezo1 localization to enlarged adhesions at the cell periphery (Figure 4(a),(b)). Additionally, there was increase in Piezo1 level upon US treatment in comparison to the untreated control (Figure 4(c)), indicating that US‐mediated mechanical forces were sufficient to promote Piezo1 expression and to promote its localization to peripheral adhesions. Next, to test the effect of nocodazole‐mediated contractility on Piezo1 localization, MDA‐MB‐231 cells were treated with nocodazole. Nocodazole‐treated cells had fewer microtubules and enlarged adhesions with no signs of Piezo1 localization to the adhesions (Figure 4(a),(b)), indicating that nocodazole‐mediated increase in contractility was not sufficient to cause Piezo1 localization to adhesions, whereas US treatment was.

To determine the role of myosin IIA contractility in Piezo1 localization, myosin IIA knock down (KD) cells were treated with the US (Figure 4(d)). Surprisingly, myosin IIA KD inhibited Piezo1 localization to the peripheral adhesions following US treatment (Figure 4(a), (b)), highlighting the role of US‐mediated contractility in regulating Piezo1 localization. Also, US treatment caused no microtubule disassembly in both myosin KD and Piezo1 KD cells (Figure 4(e)–(g)), which indicated that myosin contractility‐mediated Piezo1 localization and US activation caused calcium uptake to trigger calpain‐induced microtubule disruption. In support of these findings, we observed ~50% cells expressing Piezo1 at the cell periphery upon US treatment in comparison to 16% non‐treated control cells (Figure 4(h)). Thus, these results indicated that the US‐mediated forces indirectly activated myosin contractility to promote Piezo1 expression and its localization to the peripheral adhesions.

### 2.5 Myosin IIA contractility and Piezo1 are required for US‐induced apoptosis

**FIGURE 5:**
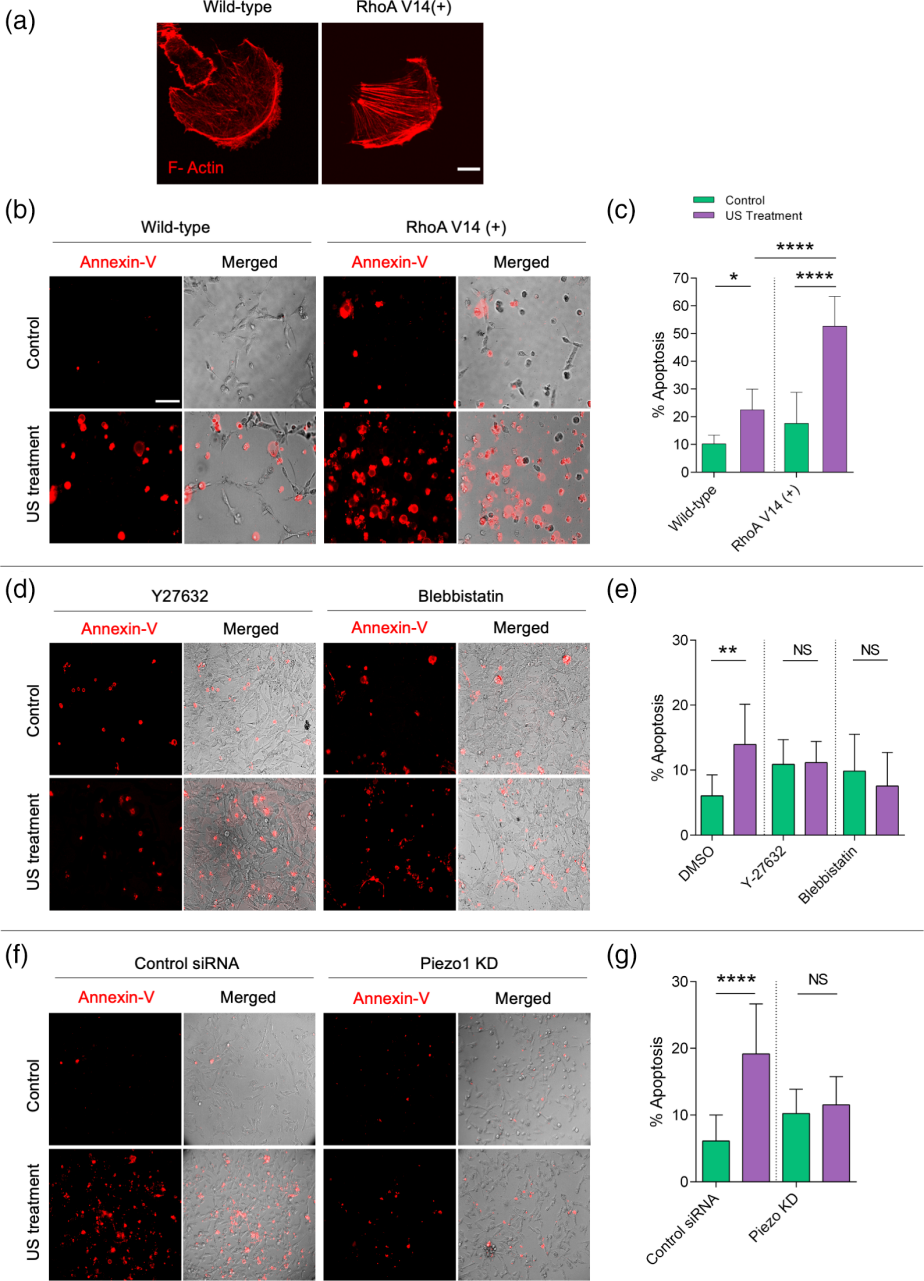
Myosin IIA contractility and Piezo1 are necessary to initiate ultrasound‐mediated apoptosis in MDA‐MB‐231 cancer cells. (a) F actin‐stained images of wild‐type and constitutively active RhoA transfected cells, scale bar: 100 μm. (b) Annexin‐stained images showing apoptotic cells in control and RhoA transfected cells with and without US treatment. (c) Bar diagram displaying percentage of apoptotic cells in control and RhoA transfected cells upon US treatment, *n* > 500 cells. (d) Annexin‐stained images showing apoptotic cells in Y27362‐ and blebbistatin‐treated cells with and without US treatment. (e) Bar diagram displaying percentage of apoptotic cells in Y27362‐ and blebbistatin‐treated cells upon US treatment, *n* > 2500 cells. (f) Annexin‐stained images showing apoptotic cells in control siRNA and Piezo1 KD cells with and without US treatment. (g) Bar diagram displaying percentage of apoptotic cells in control siRNA and Piezo1 KD samples upon US treatment, *n* > 2000 cells. *p < 0.05, **p < 0.01, ****p < 0.0001. In image panels b, d, and f, scale bar: 75 μm

These results and previous findings indicated that myosin IIA contractility was required for Piezo1 localization and activation.^27^, ^28^ Hence, we hypothesized that both myosin IIA contractility and Piezo1 were required for US‐mediated mechanoptosis. To test this hypothesis, MDA‐MB‐231 cells were transiently transfected with constitutively active RhoA (RhoA V14) to enhance the cell contractility (Figure 5(a)). US treatment caused a significantly higher level of apoptosis in RhoA expressing cells (52%) in comparison to the non‐treated RhoA expressing cells (18%) and US‐treated wild‐type cells (22%) (Figure 5(b),(c)). To further confirm the role of myosin contractility in mechanoptosis, cells were treated with contractility inhibitors, Y27632 (35 μM, ROCK inhibitor) and blebbistatin (50 μM, Myosin IIA inhibitor). US treatment did not increase apoptosis levels in the contractility inhibitor‐treated cells above the non‐treated control cells (Figure 5(d),(e)). These observations were in line with our previous results in which blebbistatin treatment inhibited the mechanical stretch‐mediated tumor cell apoptosis.^10^

Next, to establish the need for Piezo1 in the US‐mediated mechanoptosis, Piezo1 was knocked down prior to US treatment. A low level of apoptosis was observed in Piezo1 KD cells after US treatment that was comparable to non‐treated Piezo1 KD cells (~10%) (Figure 5(f),(g)), whereas cells treated with control siRNA showed an elevated level of apoptosis upon US treatment (18% vs. 5% controls). These results were in line with our previous findings.^9^ Thus, both myosin IIA contractility and Piezo1 played critical roles in US‐mediated mechanoptosis.

## 3 DISCUSSION

Although tumor cells are consistently killed by US treatment alone, most of the cell‐cycle phase inhibitors have little effect on level tumor cell killing with US treatment. Only nocodazole and vincristine (both MDAs) cause significantly higher apoptosis when combined with US treatment. In addition, we observe that US‐mediated microtubule depolymerization depends on calcium‐activated calpains and Piezo1 channels. A consequence of microtubule depolymerization is the activation of RhoA kinase pathway by the release of GEF‐H1 that promotes myosin IIA contractility. Since inhibition of myosin contractility inhibits US‐induced mechanoptosis, it is likely that the increase in myosin contractility causes an increase in apoptosis with US treatment. Also, US treatment causes an increase in Piezo1 expression and its localization to peripheral adhesions.

The US‐based apoptosis of tumor cells is consistent with several studies, which report that tumor cells are sensitive to external mechanical stresses.^5^, ^6^, ^7^, ^12^ In a process that may be related, there is growing evidence that physical exercise inhibits tumor growth in mice and humans.^6^, ^7^, ^8^ There is currently no clear understanding of how mechanical stresses are causing an increase in cytoplasmic calcium that activates tumor cell apoptosis. The power intensity of US used here is significantly low (7.7 mW/cm^2^) and does not cause mechanical damage to normal cells/tissues. Further, similar expression levels of Piezo1 are observed in many matched pairs of tumor and normal cells,^10^ but it serves different roles in the tumor and normal cells. Transformed tumor cells differ from normal cells in many aspects, since the levels of over 700 mRNAs are altered when normal cells become transformed.^3^ The “transformed cell state” may be related to the “activated wound‐healing state” and hence tumor growth is likely the result of a failure of wound‐healing cells to revert to the normal growth.^29^ Tumor cells appear softer; however, they exert higher forces on the matrices and exhibit the transformed growth on soft surfaces. It may be due to fundamental alterations in the cytoplasmic architecture of tumor cells that result in an inability to regulate calcium transients unlike the normal cells.^9^, ^10^

One of the important changes appears to be an alteration in the intracellular cytoskeletal organization of tumor cells. The higher forces that transformed cells exert on matrices also indicate that there is an altered regulation of myosin contractility. The further increase in mechanoptosis with nanomolar concentrations of MDAs appears to also involve an increase in myosin contractility downstream of Rho kinase activation by GEF‐H1. The downstream pathways activated by microtubule disruption involve the GEF‐H1 that couples microtubule depolymerization to Rho GTPase activation.^30^ Thus, the greater activation of apoptosis may due to higher microtubule depolymerization as a result of synergy between the effects of US and MDAs. In particular, Piezo1 channels may activate microtubule disruption through a calcium‐dependent katanin breakage of microtubules^31^ or through calcium‐activated calpain protease causing proteolytic degradation of microtubules.^24^, ^25^ Moreover, we find an increase in expression levels of Piezo1 in mechanically‐stressed tumor cells,^9^, ^10^ which would synergize with elevated level of calpains in tumor cells.^32^ In fact, high expression of calpain 2 in breast tumor patients is associated with negative outcome of survival.^33^, ^34^ Hence, it is reasonable to assume that an elevated level of calpains and the mechanically activated expression of Piezo1 in tumor cells make them more susceptible to the US‐mediated mechanical stresses as compared to the normal cells.

The localization of Piezo1 to the periphery of tumor cells after US treatment correlated with an increase in the calcium entry. Although, nanomolar concentrations of MDAs generated sufficient myosin contractility to promote larger peripheral adhesion formation, they did not affect Piezo1 expression and distribution. However, with US treatment, a coordinated feedback loop activated in which increased myosin contractility enhances Piezo1 expression, its localization to cell periphery and subsequently activated Piezo1‐mediated calcium entry to disrupt microtubules via calpains. The presence of a feedback loop involving RhoA activation further strengthens the effects of activators or inhibitors of myosin contractility through Piezo1 expression levels. Thus, the mechanoptosis level can be toggled using myosin contractility activators (high level) and inhibitors (low level). Since this feedback process involves changes in Piezo1 expression, it involves tens of minutes and requires prolonged exposure to the US treatment or mechanical stretch.

**FIGURE 6:**
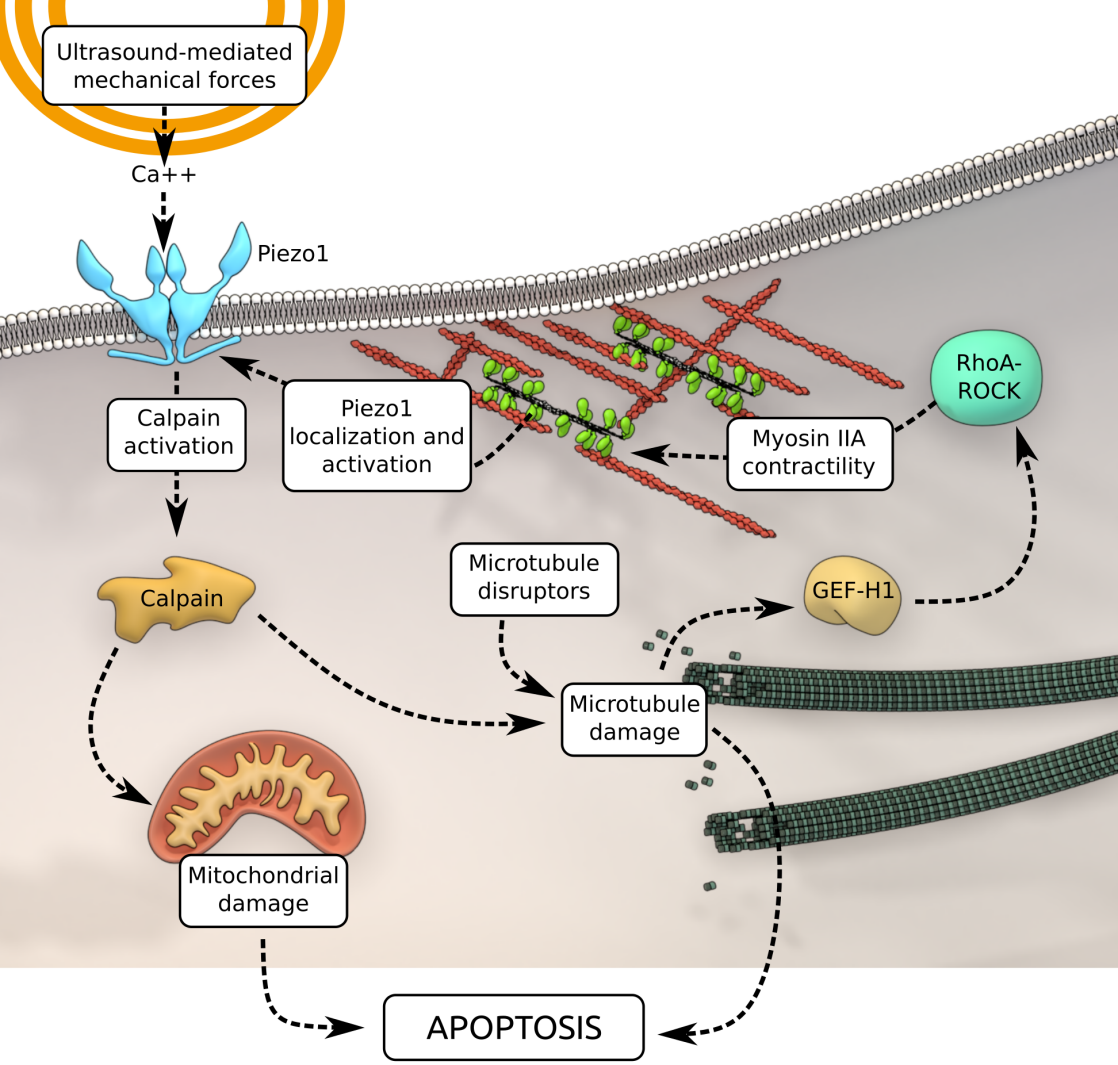
Microtubule depolymerizing agents (MDAs) augment ultrasound‐mediated tumor cell apoptosis. Ultrasound‐mediated mechanical forces initiate calcium entry through Piezo1 channels. Calcium‐activated calpain triggers mitochondria‐dependent apoptotic pathway. Also, calpain disrupts microtubules to enhance myosin IIA contractility through RhoA pathway activation. Cell contractility promotes Piezo1 localization to cell periphery and their activation to continue the feedback loop. In addition, MDAs by disrupting the microtubule network further potentiate tumor cell mechanoptosis

Based upon these findings, we proposed that US‐mediated mechanical forces and microtubule disruptors have a synergistic role in augmenting the tumor cells apoptosis (Figure 6). US‐mediated mechanical forces activate Piezo1 to allow calcium uptake inside the cell. As described previously, calcium‐activated calpains trigger apoptosis through a mitochondria‐dependent pathway.^10^ Concomitantly, calpains disrupt microtubule assembly to release GEF‐H1, which in turn enhances myosin contractility through RhoA activation. Myosin contractility promotes Piezo1 expression and its localization to the peripheral adhesions. Elevated levels of Piezo1 at cell periphery further cause increased calcium influx to continue the feedback loop. In addition, MDAs disrupt microtubules to further activate RhoA pathway to enhance the apoptosis.

Since clinically approved MDA, vincristine is widely used to treat multiple sarcoma (rhabdomyosarcoma and Kaposi's sarcoma)^35^, ^36^, ^37^ and our recent results showed that US treatment selectively killed fibrosarcoma cells without damaging normal fibroblasts,^9^ we speculate that the combined therapy of MDA and US can be exploited clinically for sarcoma treatment.

## 4 CONCLUSION

In summary, we showed that US‐mediated mechanical forces caused microtubule disruption in tumor cells. Molecular mechanistic study revealed that US initiated the calcium entry through Piezo1 channels that disrupts microtubules via calpain protease activation. Myosin contractility was necessary for US‐mediated tumor cell apoptosis and microtubule disruption enhanced myosin contractility through activation of GEF‐H1 and RhoA pathway. Also, US treatment promoted Piezo1 expression and its localization to the peripheral adhesions where activated Piezo1 allowed calcium entry to continue feedback loop. Collectively, these findings open a new avenue for tumor treatment that should reduce drug‐related side effects by increasing the level of apoptosis with US treatment in the presence of clinically approved MDAs.

## 5 MATERIALS AND METHODS

### 5.1 Cell culture

Cell lines MDA‐MB‐231 (gift from Dr. Jay Groves, UC Berkeley), A375p (gift from Dr. Chris Bakal, ICR UK), and HFF (ATCC) were cultured in high glucose DMEM (ThermoFisher) supplemented with 10% FBS and 1% penicillin and streptomycin at 37°C in 5% CO_2_ incubator. MCF10A, human breast epithelial cells (ATCC) were cultured in a specialized culture medium as mentioned previously.^38^ For cell experiments, high‐glucose DMEM containing 10% FBS and 1% penicillin and streptomycin was utilized. For cell trypsinization, TrypLE (ThermoFisher) was used. For apoptosis assay, cells were cultured on matrigel‐coated 96 well plates (Corning). For high‐resolution imaging, cells were grown on fibronectin‐coated glass bottom dishes. Ten thousand cells per well were seeded and treated with pharmacological drugs overnight after 4 h post‐cell attachment.

### 5.2 Surface coating with matrigel

Ninty‐six well plate surface was coated with thin layer of matrigel (Corning) according to manufacturer's protocol. Briefly, matrigel (Corning) diluted with cold PBS (1:2) (33 μl) was quickly added on pre‐chilled surface of a single well of 96 well plate. Well plates were then incubated at 37°C for 24 h to solidify matrigel before performing cell seeding experiments.

### 5.3 Plasmid transfection

For transient transfection of mApple‐paxillin and constitutively active RhoAV14, Lipofectamine 3000 reagent (ThermoFisher) was used according to the manufacturer's protocol.

### 5.4 siRNA assay

Cells were cultured in the six well plate. Calpain 1, calpain 2, Piezo1 (Dharmacon), myosin IIA (against MYH9, Sigma), and scramble siRNA (Sigma) transfection were performed on next day using lipofectamine RNAiMAX (Invitrogen) according to the manufacturer's protocol. Calpain siRNAs were fabricated by cloning facility MBI, Singapore, as described before.^10^

### 5.5 Apoptosis assay

To identify cell apoptosis, Annexin V‐Alexa Fluor 488 or Annexin V‐Alexa Fluor 594 conjugates (Thermofisher) were used according to the manufacturer's protocol. Assay was performed 12 h after US treatment.

### 5.6 US treatment

The details of customize‐built US device fabrication were mentioned previously.^9^ Prior to ultrasonication, cells were treated with the cell‐cycle inhibitors for 16 h with cytostatic concentrations as stated in Table S1. Drug containing culture medium was then replaced with fresh culture medium. Well plates/dishes were quickly sealed with parafilm to avoid possible contamination and water entry during US treatment Samples were then placed 8 cm above transducer in the US tank, which was mounted in the incubator with 37°C. Tank was filled with DI water to half submerge the samples. Samples were then treated with 33 kHz frequency for 2 h with 50% duty cycle. Samples were returned to the standard incubator for overnight incubation to determine the apoptosis level on following day.

### 5.7 Immunocytochemistry and fluorescence microscopy

Cells were fixed with either chilled 100% methanol for 10 minutes or 4% paraformaldehyde solution at room temperature for 15 min and subsequently permeablized with 0.2% Triton‐X in PBS for 5 min. Cells were blocked for 1 h using 2% normal goat serum in PBS prior to overnight incubation with primary antibody solution at 4°C: mouse monoclonal anti‐alpha tubulin (Sigma, catalogue no. T6199, 1:400), rabbit polyclonal anti‐paxillin (Abcam, catalogue no. ab191007, 1:200), rabbit polyclonal anti‐GEF‐H1 (Abcam, catalogue no. ab155785, 1:200), rabbit polyclonal anti‐myosin IIA (Sigma, catalogue no. M8064, 1:200), and rabbit polyclonal anti‐Piezo1 (Novus Biologicals, catalogue no. NBP1‐78446, 1:200). Samples were washed with PBS three times and incubated with Alexa Fluor‐488 or −594 secondary antibodies (ThermoFisher) for 1 h at room temperature followed by PBS washing. F‐actin was stained using Alexa Fluor 594 phalloidin conjugated antibody (ThermoFisher). Cell nucleus was stained using Hoechst dye (1:1000, ThermoFisher).

Images were acquired using either wide‐field Olympus live‐EZ microscope equipped with photometrics CoolSNAP K4 camera or W1 live‐SR spinning disk microscope equipped with photometrics Prime 95B sCMOS camera.

### 5.8 Western blot study

Cells were incubated with siRNA for 48 h, pelleted, washed with PBS. Cell lysis was done using RIPA buffer (Sigma) supplemented with 1X cOmplete protease inhibitor (Roche, catalogue no. 4693116001). Cell lysate was centrifuged at 15,000 revolutions per minute, 4°C for 20 min. Supernatant was mixed with 2X loading dye (2X Lammeli Buffer, Bio‐Rad, catalogue no. 1610737) + beta‐Mercaptoethanol (Sigma). Samples were denatured for 5 min at 95°C and gel was run using a 4%–20% Mini‐PROTEAN® TGX precast protein gels (Bio‐Rad, catalogue no. 4561094). Gels were then transferred onto membrane and blocked with 5% BSA solution in 1X TBST (Tris‐Buffered Saline with Tween‐20) for 1 h. Next, membrane was incubated with primary antibody overnight at 4°C on shaker. Membranes were washed with TBST three times (10 min per wash) followed by incubation with secondary antibodies in 1X TBST (Horse Radish Peroxidase –HRP) for 1 h. The chemiluminescence of the membranes was developed using Super Signal Femto Substrate Kit (Pierce) and developed using ChemiDoc Touch Imager (Bio‐Rad). Primary antibodies and conditions used are as follow: Calpain 1 (rabbit, 1:1000, Abcam, ab39170), calpain 2 (rabbit, 1:1000, Abcam, ab39165), Piezo1 (rabbit, 1:500, Novus Biologicals, NBP1‐78446), alpha‐tubulin (mouse, 1:3000, Sigma T9026), and rabbit polyclonal anti‐myosin IIA (Sigma, catalogue no. M8064, 1:1000). Secondary antibodies are as follow: HRP‐conjugated goat anti‐rabbit IgG (1:2000, Bio‐Rad, 170‐6515) and goat anti‐mouse IgG (1:2000, Bio‐Rad, 170‐6516).

### 5.9 Pharmacological inhibitor study

Pharmacological inhibitors of cell cycle were used as follow: Binimetinib (1 μM), Palbociclib (2 μM), MK‐8776 (1 μM), AZD‐7762 (1 μM), RO‐3306 (9 μM), Roscovitine (1 μM), Nocodazole (50 nM), Paclitaxel (10 nM), and Vincristine (10 nM). Cell‐cycle inhibitors were procured from Selleckem. All inhibitors were reconstituted with DMSO, except vincristine with DI water as recommended by the manufacturer. Cells were treated with drugs overnight after 4 h post‐cell attachment.

Other pharmacological inhibitors were used as follow: ALLN (50 μM, Sigma), Blebbistatin (50 μM, Sigma), and Y‐27632 (35 μM, Sigma). These inhibitors were added to culture medium an hour post‐cell seeding and US treatment was initiated an hour after addition of inhibitors.

### 5.10 Microtubules analysis

Assessment of extent of microtubule network disruption upon US treatment was manually quantified per 10 μm^2^ area, taking into consideration an entire uninterrupted strand extending toward cell periphery as one. Strands forming any cross‐linked intersections were scored as two. Strands observed as diffused structures with fluorescent dots undergoing severe microtubule disruption were scored as zero.

### 5.11 Statistical analyses

GraphPad Prism version 6 was used to plot and analyze the data. Statistical analysis was carried out using one‐way ANOVA unless otherwise stated, considering *p‐*value <0.05 as statistically significant. The quantitative data represented as bar graphs or box‐and‐whisker plots were shown as mean ± standard deviation. The whiskers in box‐and‐whisker plots extend from the minimum to maximum values, the box extends from the 25th to the 75th percentile, and the line within the box represents the median.

## AUTHOR CONTRIBUTIONS

**Aditi Singh:** Data curation; formal analysis; investigation; validation; visualization; writing‐original draft. **Ajay Tijore:** Conceptualization; data curation; formal analysis; investigation; supervision; visualization; writing‐original draft. **Felix Margadant:** Methodology; validation. **Chloe Simpson:** Methodology; validation. **Deepak Chitkara:** Supervision. **Boon Chuan Low:** Funding acquisition; project administration; supervision. **Michael Sheetz:** Conceptualization; funding acquisition; supervision; writing‐original draft.

## CONFLICT OF INTERESTS

Ajay Tijore, Felix Margadant, and Michael Sheetz have interest in a company, Mechanobiologics, Inc. that is involved in developing mechanical therapies for cancer and aging.

### PEER REVIEW

The peer review history for this article is available at https://publons.com/publon/10.1002/btm2.10233.

## Supporting information

**Appendix S1.** Supporting Information.

## Data Availability

The raw data of study will be available from the corresponding authors upon request.
