# Supplementary material for: Enhanced tumor cell killing by ultrasound after microtubule depolymerization
Source: Bioeng Transl Med. 2021 Jun 11;6(3):e10233. doi: 10.1002/btm2.10233 (PMC8459596; doi:10.1002/btm2.10233)
Supplement: Supplementary file 1 — Appendix S1. Supporting Information. [file BTM2-6-e10233-s001.docx]

**Supplementary Data**

**Enhanced tumor cell killing by ultrasound after microtubule depolymerization**

Aditi Singh^†1,2^, Ajay Tijore^†1^*, Felix Margadant^1^, Chloe Simpson^1^, Deepak Chitkara^2^, Boon Chuan Low^1^, Michael Sheetz^1,3^*

1Mechanobiology Institute, National University of Singapore, Singapore

^2^ Department of Pharmacy, Birla Institute of Technology and Science, Pilani, India

^3^Biochemistry and Molecular Biology Department, University of Texas Medical Branch, Galveston, Texas, USA

| **Inhibitor** | **Cell cycle phase** | **Working conc.** | **Mode of action** |
| --- | --- | --- | --- |
| Binimetinib | G1 | 1 µM | MEK 1/2i |
| Palbociclib |  | 2 µM | CDK 4/6i |
| MK-8776 | S | 1 µM | CHK1i |
| AZD-7762 |  | 1 µM | CHK 1/2i |
| RO-3306 | G2 | 9 µM | CDK1i |
| Roscovitine |  | 1 µM | CDK 1/2/5/7i |
| Nocodazole | M | 50 nM | Microtubule depolymerization |
| Vincristine |  | 10 nM | Microtubule depolymerization |
| Paclitaxel |  | 10 nM | Microtubule polymerization |

**Table S1**. Pharmacological inhibitors to target different cell cycle phases using their cytostatic concentration.


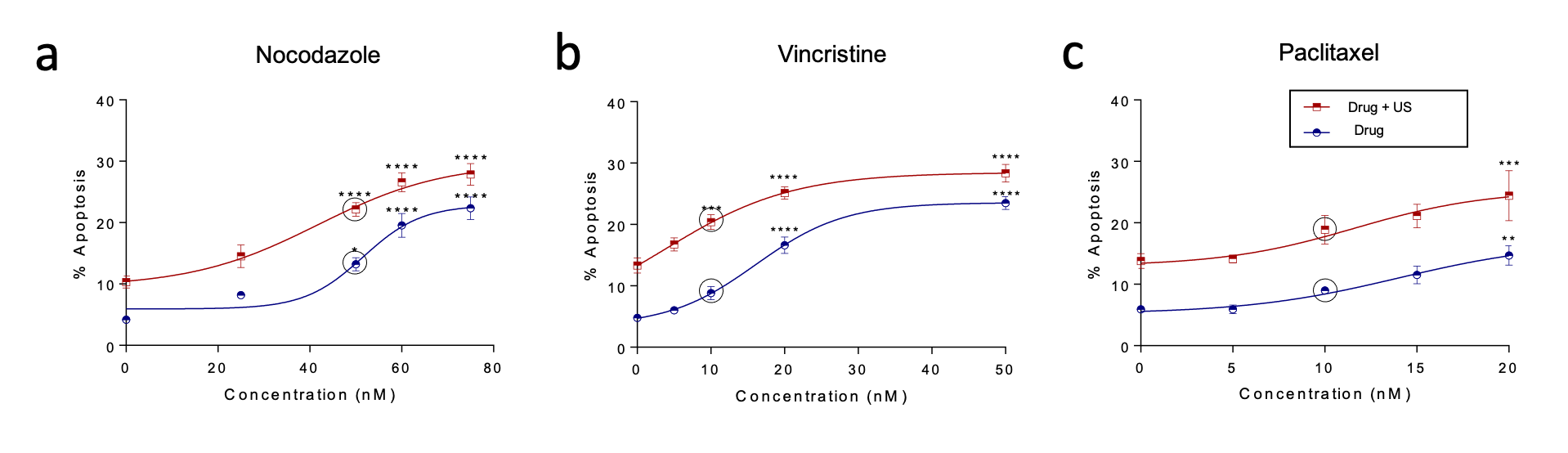


**Figure S1.** (a-c) Dose response curves for MDA MB-231 cells using nocodazole, vincristine and paclitaxel. Data is represented with errors in ± S.E.M. and curves are best fitted with non-linear fit models with 4 parameters. Encircled values represent the optimum cytostatic concentrations of the drugs used for experiments. One-way Tukey’s ANOVA was performed to determine significance of apoptosis for each drug concentrations with respect to their controls.


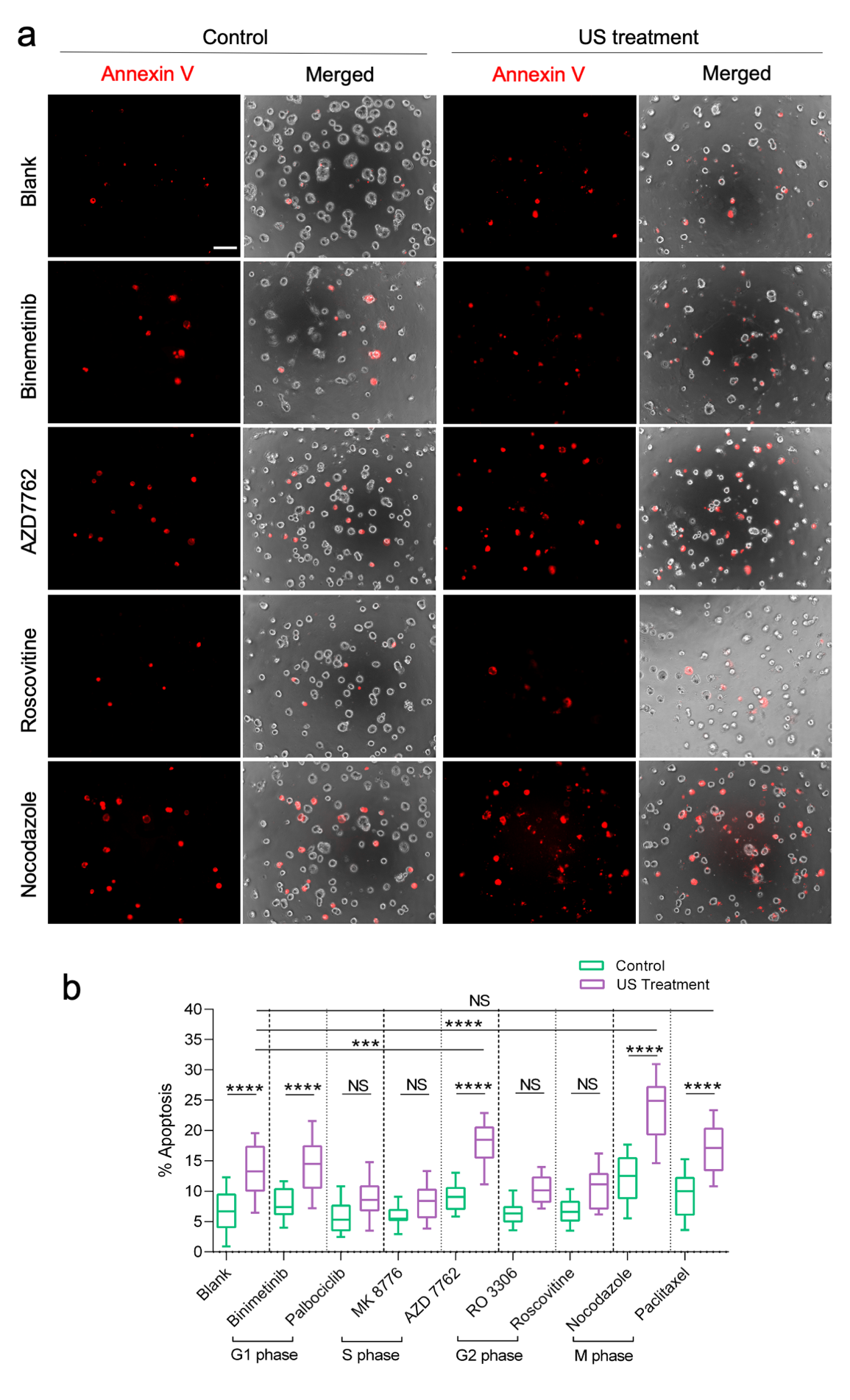


**Figure S2.** Microtubule depolymerizing agent (MDA) promotes ultrasound-mediated apoptosis in A375p melanoma cells. (a) Panels showing annexin V-stained apoptotic cells under different conditions, scale bar: 100 µm. (b) Bar diagram displaying level of apoptosis in the presence of various pharmacological inhibitors with and without US treatment. n>1000 cells, data are representative of three independent experiments. ****P*<0.001, *****P*<0.0001.


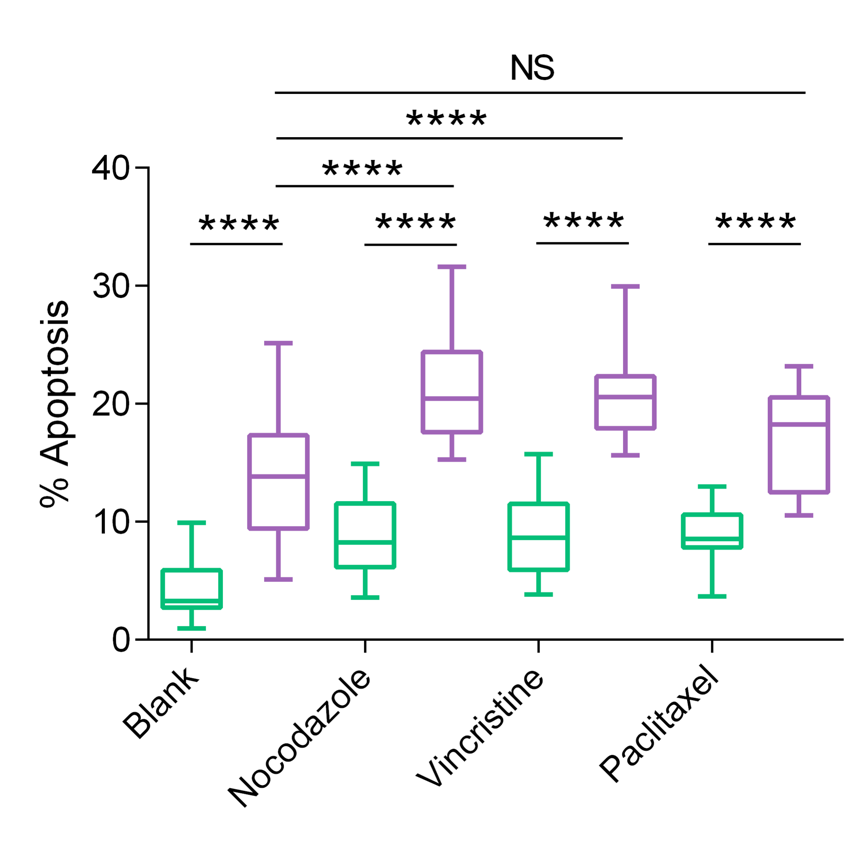


**Figure S3.** MDAs enhance ultrasound-mediated apoptosis in MDA-MB-231 tumor cells. Box and whiskers plot showing level of apoptosis in the presence of various microtubule targeting agents with and without US treatment. n>1500 cells, data are representative of three independent experiments. *****P*<0.0001.


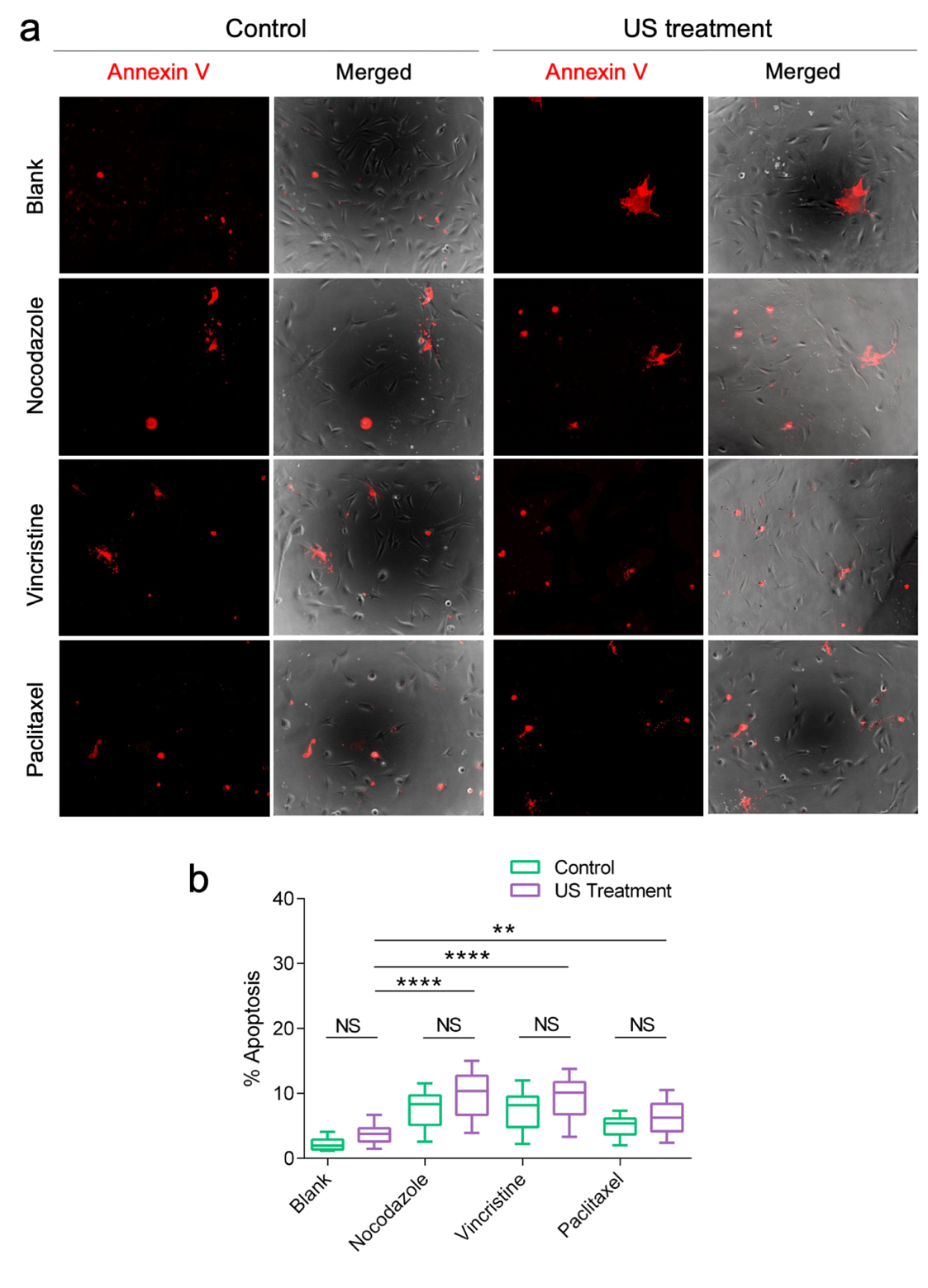


**Figure S4.** Primary fibroblasts (HFFs) remain unaffected by ultrasound- and MDA-mediated apoptosis. (a) Panels showing annexin V-stained apoptotic fibroblasts under different conditions, scale bar: 50 µm (b) Box and whiskers plot illustrating level of apoptosis in the presence of microtubule targeting agents with and without US treatment. n>1000 cells, data are representative of three independent experiments. ***P*<0.01, *****P*<0.0001.


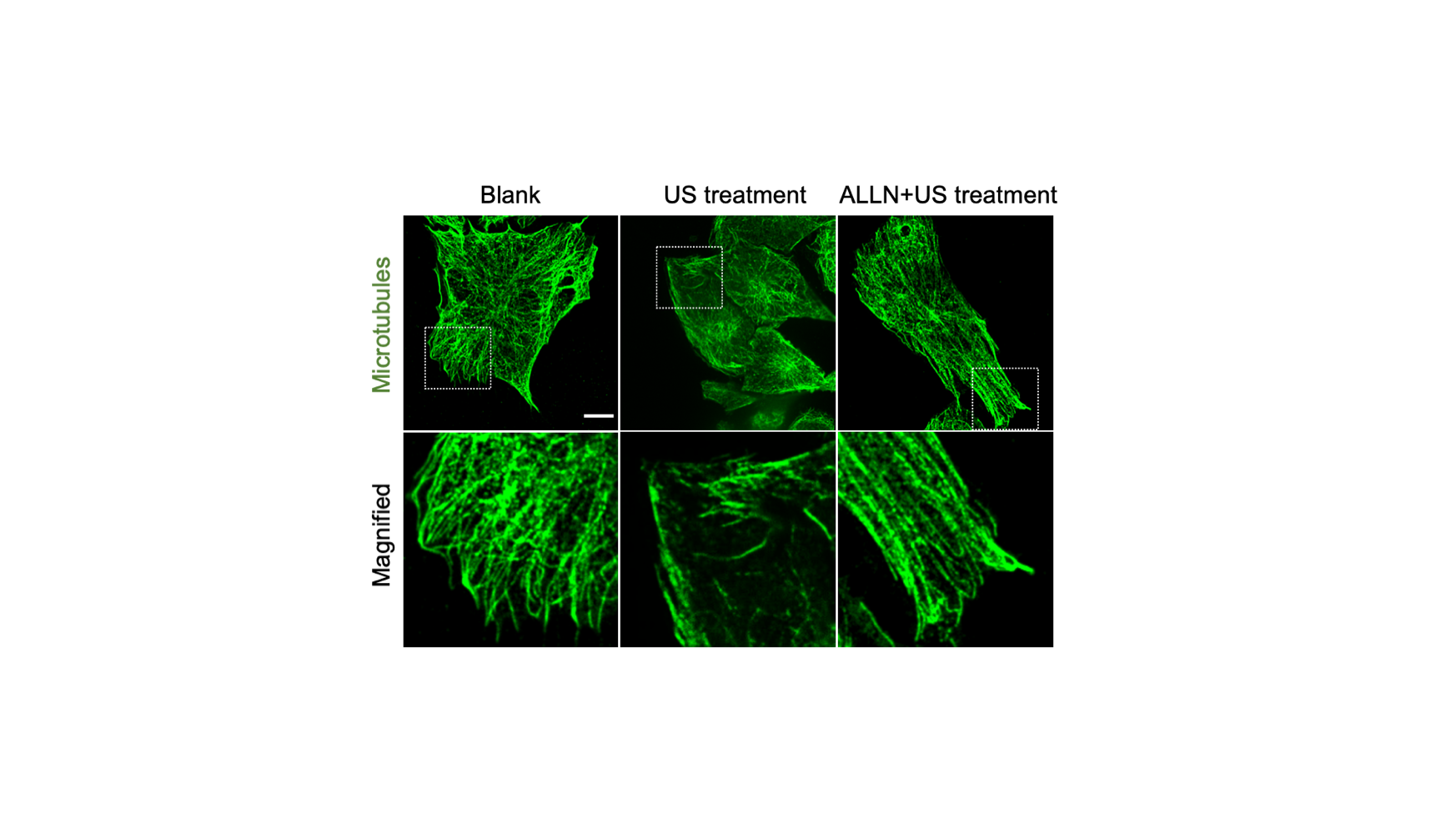


**Figure S5.** Calpain is responsible for microtubule disruption. Panels showing microtubule assembly in the control (blank), US treated and ALLN+US treated MDA-MB-231 tumor cells. Scale bar: 10 µm.
